# OFFl Models: Novel Schema for Dynamical Modeling of Biological Systems

**DOI:** 10.1371/journal.pone.0156844

**Published:** 2016-06-07

**Authors:** C. Brandon Ogbunugafor, Sean P. Robinson

**Affiliations:** 1 Department of Organismic and Evolutionary Biology, Harvard University, Cambridge, MA, United States of America; 2 Department of Physics, Massachusetts Institute of Technology, Cambridge, MA, United States of America; SUNY Downstate MC, UNITED STATES

## Abstract

Flow diagrams are a common tool used to help build and interpret models of dynamical systems, often in biological contexts such as consumer-resource models and similar compartmental models. Typically, their usage is intuitive and informal. Here, we present a formalized version of flow diagrams as a kind of weighted directed graph which follow a strict grammar, which translate into a system of ordinary differential equations (ODEs) by a single unambiguous rule, and which have an equivalent representation as a relational database. (We abbreviate this schema of “ODEs and formalized flow diagrams” as OFFL.) Drawing a diagram within this strict grammar encourages a mental discipline on the part of the modeler in which all dynamical processes of a system are thought of as interactions between dynamical species that draw parcels from one or more source species and deposit them into target species according to a set of transformation rules. From these rules, the net rate of change for each species can be derived. The modeling schema can therefore be understood as both an epistemic and practical heuristic for modeling, serving both as an organizational framework for the model building process and as a mechanism for deriving ODEs. All steps of the schema beyond the initial scientific (intuitive, creative) abstraction of natural observations into model variables are algorithmic and easily carried out by a computer, thus enabling the future development of a dedicated software implementation. Such tools would empower the modeler to consider significantly more complex models than practical limitations might have otherwise proscribed, since the modeling framework itself manages that complexity on the modeler’s behalf. In this report, we describe the chief motivations for OFFL, carefully outline its implementation, and utilize a range of classic examples from ecology and epidemiology to showcase its features.

## Introduction

When faced with the collected observations of a natural system, one of the principal tasks for a scientist is to bring some level of understanding or order to the observations, typically by collating the raw facts according to some conceptual framework, a theory. The framework could be an existing paradigm for describing broad categories of observations with a minimum of principles, or a novel model meant only to capture the quantitative details of a single study, or somewhere in between [1].

The broad approach has proven especially challenging in biological and medical contexts, where the complexity of most systems precludes a reductionist interpretation of observations in terms of a few fundamental principles. In these cases, the ability to quickly generate and evaluate the space of possible models for a system is of great practical importance for advancing the scientific understanding of the system and applying that understanding to real world situations.

Unfortunately, there seems to exist a correlation between the scientific fields where complexity renders facile model building the most important and those fields whose professional culture is associated with an aversion to the most powerful language in which to express models, that is mathematics [2]. Conversations about the relative cultures of mathematics and other fields—in particular biology—have emerged in several forums in recent years. Some of them have arisen as debates in major journals, others as informal discussions taking place mostly in cyberspace [3, 4]. Part of this cultural divide involves the question of extent: while all parties might agree that mathematical tools allow useful description of systems and phenomenon, on what problems should such approaches be utilized?

The divide notwithstanding, practitioners in biological fields exhibit an impressive command of the vast natural histories (observations) of the systems they study, and the organizational schema commonly used to understand them. If there were clearer, more universal methods to describe biological processes, then cross-disciplinary translation would be easier. This implies to us that a set of tools which put the power of mathematical modeling into the hands of biological and medical practitioners—and their students—which do not require advanced mathematical training would be of great practical importance in helping to bridge the cultural divide.

Here, we describe an organizational schema which enables one to study and understand dynamical systems of the sort that commonly arise in problems of epidemiology, population growth in both human and ecological (*e.g.*, predator-prey and consumer-resource) systems, chemical kinetics, and the like. In all of these situations, parcels of various dynamical species are transformed into parcels of other species at rates determined by a set of interaction rules that depend on the population values of the species. The dynamics of such systems are well-described by rate equations which are systems of coupled (possibly nonlinearly) ordinary differential equations (ODEs). These ODE models are often referred to as deterministic compartmental models. A representative example of this general class of systems are so-called chemical reaction networks (see [5] and references therein), a term which emphasizes the underlying network character of the system. Analogously, we will refer to the more general class of systems considered here as *interaction networks*, but we will not attempt to define that term with any specific rigor beyond the above description.

The core of the schema is an organizational framework which enforces a certain mental discipline for the modeler, but which is not inherently mathematical, making it readily accessible to anyone wishing to translate practical knowledge of biological processes into the schema. The remainder of the schema is a set of sophisticated but algorithmic rules—easily automated—which translate the model into ODEs solvable by standard techniques. We emphasize that any practical implementation of the schema as a software tool could and should bury these automated rules behind a non-mathematical user interface, separating the scientific modeling process from the mathematics used to represent and solve the models. In contrast, this manuscript is addressed to those researchers who might implement such software tools or otherwise investigate the mathematical structures which underlie the schema, and who therefore require more technical detail than the would-be tool user.

To illustrate the use of this modeling approach, we will first introduce the schema, then explain its theoretical underpinnings, and finally demonstrate its utility with a few classical examples from ecology and epidemiology. In the course of this discussion we hope to make clear that the schema and its automatability empowers the modeler to consider significantly more complex models than practical limitations might have otherwise prescribed. Likewise, tools built with this schema would appear to be ideally suited for teaching dynamical modeling to the biological and medical student communities, where quantitative modeling is currently underserved [6] due to a perceived deficit in prerequisite mathematical training. Nevertheless, we do include here a technical discussion of the mathematical back end of the schema.

## Methods

### Flow diagrams, ODEs, and the modeling process

When considering systems which can be understood as interaction networks (as defined above), we would like to have a model development process that lets the modeler follow a procedure similar to the following:

1. Identify all the dynamical quantities (the different species or compartments) in the system.
2. Identify all the processes (interactions between species, be they biological, ecological, physical, or otherwise) in the system.
3. For each process, identify which quantities are “consumed” by the interaction and which quantities they are transformed into.
4. From this accounting of species and interactions (which constitutes the model of the system), move quickly to a set of ODEs that can be analyzed by standard means, either analytically or numerically.

At an abstract level, the above steps describe a large range of specific modeling techniques already in common use across a variety of fields.

**Fig 1:**
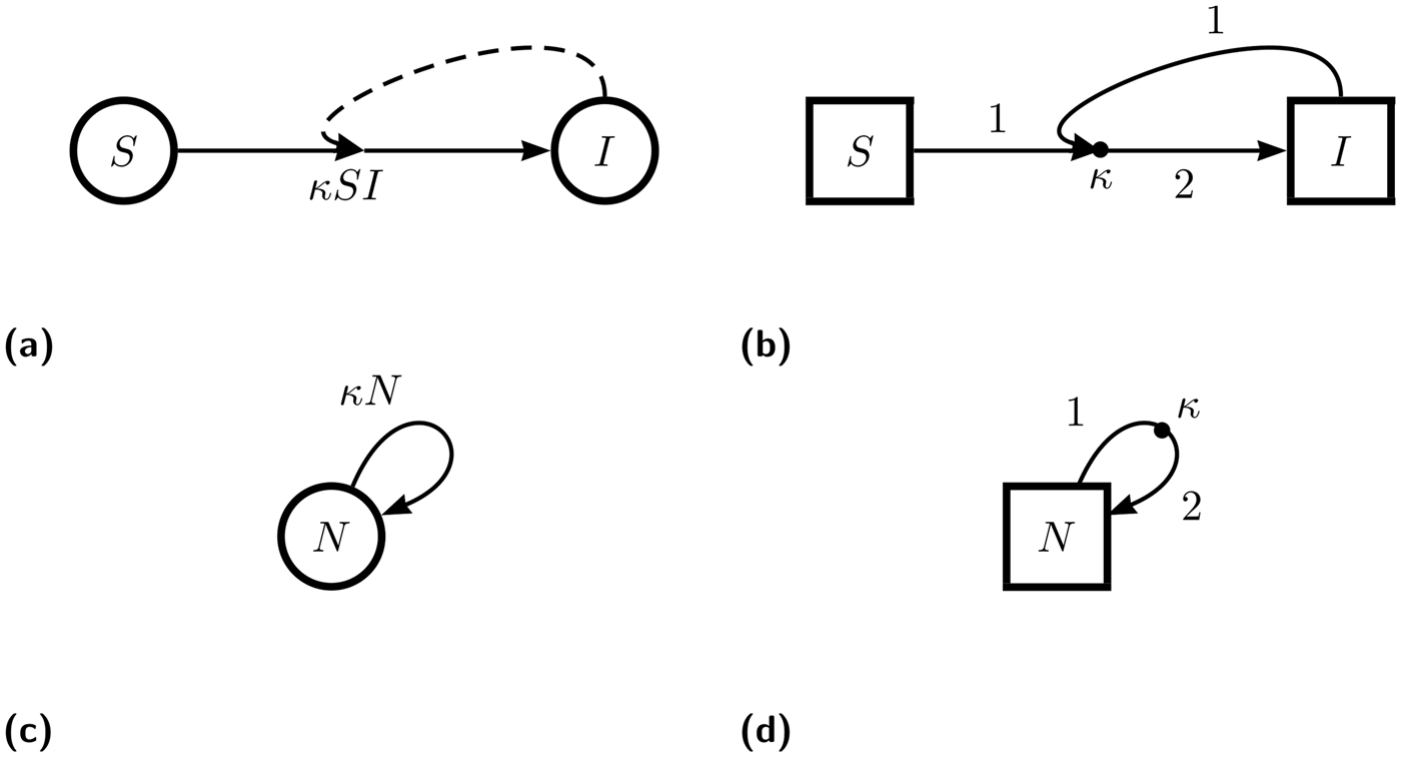
Examples of flow diagrams from two different diagrammatic schema. Diagrams (a) and (b) both represent a simple consumer-resource interaction between a “susceptible” species *S* and an “infectious” species *I*—such as is used in the simplest SI (susceptible-infected) epidemiological models—but do so in different diagrammatic schema. The edge labels in diagram (b) further specify a model in which one parcel each of *S* and *I* combine to form two parcels of *I*. Both diagrams resolve to the ODE system *dI*/*dt* = −*dS*/*dt* = *κSI*. Diagrams (c) and (d) both represent exponential growth for a population of size *N*, but do so in different diagrammatic schema. The edge labels in diagram (d) further specify a model in which each parcel of *N* splits to form two parcels of *N*. Both diagrams resolve to the ODE *dN*/*dt* = *κN*. Diagrams (a) and (c) are depicted with the widely used schema of [7], conceptually representing different kinds of positive feedback processes, while diagrams (b) and (d) use the schema described in this document (“OFFL”, defined below) and formally represent the routing of parcels of the population through interaction processes.

One such technique is the use of “flow diagrams” or “box diagrams”, shown by example in Fig 1. As described in a variety of sources (for example [7] or similar textbooks) flow diagrams have been of enormous practical use in epidemiology and ecology, but their utility is more general than these fields. In this technique, each species is written as a small box with the name (or variable name) of the species in it, and directed arrows show the flow of the interactions from one species to another. Individual modelers may apply the technique differently from problem to problem, using it as an informal method for keeping one’s thoughts organized.

One example of a systematized implementation of flow diagrams is that given by the textbook of Otto and Day [7], in particular Boxes 2.3, 2.4, and 2.5. In this implementation, the arrows are labeled with the functional form of the interaction rate. In passing from the diagram to ODEs, different interpretive rules are used for arrows which connect species versus arrows that loop back to their source, as in Fig 1c. Also, different symbology must be used to indicate arrows that are part of qualitatively different interactions. For example, solid lines flowing from the source to the target in a consumer-resource interaction are supplemented by an additional dashed line looping from the target to the midpoint of the the solid arrow, as illustrated in Fig 1a.

One might hope that such diagrams could be interpreted loosely as networks or graphs, in the mathematical sense, such that the understanding and utilization of models described by these diagrams could benefit from the tremendous advances that have been made in network theory over the past two decades, as well as from the simultaneous proliferation of software tools now available to analyze these systems. With modern network tools, analysis of truly enormous systems could be possible [8, 9]. Indeed, to describe very complex systems of ODEs such as gene regulation networks or “all world” economy-ecology models (for example, [10]), it is imperative that any flow diagram representation be brought under strict control, if only to avoid errors in the modeling process. However, existing rule schema for constructing and interpreting these diagrams as a system of ODEs often exhibit some level of interpretational ambiguity which must be solved by human intervention, and are not as yet sufficiently regular to bring flow diagrams fully under the purview of network theory. The new organizational schema for managing ODEs and formalized flow diagrams (“OFFL”) presented in the following sections aims to alleviate this difficulty.

### Formalizing flow diagrams in the OFFL schema

To enable this level of rigor, the OFFL schema prescribes a formalized version of the modeling process described above, including the representation of interaction network systems as formalized flow diagrams. In OFFL, such systems are thought of as a directed network of species and interactions, with any given interaction having some species as sources and others as targets. (The nodes of the network represent the species and interactions while the edges represent flows of parcels from species to interactions and vice versa. This way of thinking is already a significant departure from common practice with flow diagrams. For example, it forbids edges which enter or leave the network from “nowhere”.) We therefore want the schema to force the modeler—motivated by a physical, biological, or ecological understanding of the system being modeled—to work directly at the level of the dynamical quantities under examination and the processes which affect them, rather than, say, skipping ahead to a particular differential equation or dynamical solution. Thus, the structure of the flow diagrams should reflect this low-level approach in a way that can be uniformly applied to all possible processes.

In the diagrammatic schema to be presented here, an arrow leaving a species will represent a negative contribution to that species’s rate of change while an incoming arrow will represent a positive contribution. Thus, for example, without any further adornments, an arrow that loops back into its source represents a parcel of the species leaving and then reentering the population, for no net change. This is another departure from typical usage, in which a self-looping arrow represents a positive feedback, such as in the exponential growth model shown in Fig 1c. We view this departure from convention as an advantage because even the case of simple exponential growth is not simply a feedback, but rather a process in which each parcel is amplified at each time step by a reproduction process. We would like the diagram to not just to indicate a feedback in the numerical value, but also to capture the physical cause of that feedback. So, in this case, the self-looping arrow should be interrupted by a new node: a “dot” representing the reproduction process, as in Fig 1d. The dot itself carries the rate constant of the process (literally a constant in the case of exponential growth), while its incoming and outgoing edges carry additional labels showing the proportions of how much incoming “stuff” turns into how much outgoing “stuff”.

In fact, to achieve a uniform application of the schema in a way that enforces thinking at a process level, *all* interactions between species in the network will be represented by a labeled dot connected to species by arrows. Furthermore, species will *never* be connected to each other except via an interaction. For example, in the simplest SI (susceptible-infected) epidemiological model (illustrated in Fig 1), one might informally think of the basic mechanism as following the schematic equation *S* + *I* → *I*, but being more careful, we recognize that reality is better reflected by *S* + *I* → *I* + *I* = 2*I*. (A critical insight into such systems is that the total number of people is conserved in every encounter!) The former way of thinking about the system is captured in the flow diagram of Fig 1a, while the latter way of thinking, which we believe to be more disciplined and rigorous, is expressed in Fig 1b.

Therefore, in OFFL, the SI model shows the infection process as a dot—labeled with a rate constant—which draws in parcels from two source species and then outputs parcels to a target species with twice the “weight”. In contrast, Fig 1a simply shows one species flowing directly to the other, representing the infection process as an arrow. The additional dashed line in Fig 1a is cosmetic, indicating to the reader that the rate of the process is modulated by another species. This point of departure from typical usage of flow diagrams is worth repeating: in an OFFL diagram, both species and processes will be represented as nodes of the network while an edge represents a relationship between a species and an interaction, whereas in typical diagrammatic schema, only species appear as nodes while edges represent processes.

The labels on the edges and on the interaction process together contribute to a rule which governs the rate of change for each species involved in the process: how much to take away from each source species and how much to redistribute to each of the targets. The sum of these contributions to each species over all processes constitutes the dynamics of the system, expressed as a set of ODEs. How one moves from a descriptive understanding of the system to a formal representation as a flow diagram and then to ODEs is the subject of the next several sections of this manuscript.

It is worth noting that rates of interaction—if left to chance—depend on the rate of uncorrelated random encounters between parcels of the source species. Therefore, a given interaction’s contribution to the system dynamics will be proportional to the value of each of its source species (not the target species), as well as having a contribution inherent to the interaction itself. For example, in SI infection models, the rate of new infection is proportional to *SI* and additionally proportional to a rate constant describing how often *S* and *I* encounters which lead to new infection occur. This proportionality is part of the kinematic structure of such models—a mass action law arising from an underlying dynamics in the uncorrelated microscopic degrees of freedom, consisting of a random walk or diffusion with localized “billiard ball” interactions. Deviations from this proportionality reflect interesting dynamics of the interaction process beyond that of simple randomly walking parcels. (For example, transmission rates of sexually transmitted diseases or predation rates in ecological systems with ample prey are not simply proportional to the values of the involved populations because the interaction event itself can only occur a maximum number of times in any fixed time period.) Given this, and given that we want the flow diagram to distinguish the modeling of the interaction process from the basic kinematic structure of population dynamics, the label on an interaction process in an OFFL diagram should specify the fractional rate of transformation of the source species (*e.g.*, per capita change per hour) such that the absolute transformation rate (*e.g.*, quantity per hour) which contributes to the final ODEs will be given by the product of this fractional rate with the population values of each source species.

### OFFL schema: from a system model to a diagram

Based on the above discussion of general principles, we now propose a specific process for developing a model and representing it as a flow diagram as follows. (Terms introduced as jargon of the schema are indicated in *italic* typestyle. They are collected and defined in S1 Appendix.)

1. Think carefully about the system, about which features are important for modeling and which can be ignored, and about how the important features can be quantified as measurable numbers. Decide which aspects of the system are dynamical quantities—that is, changing in time in response to the values of the other dynamical quantities—and which aspects are the processes that cause those dynamical changes. (The dynamical quantities of the system will be represented by the species of the model. The processes of the system will be represented by the interactions of the model.)
2. Consider each process to be drawing *parcels* from certain dynamical quantities at some rate, transforming them, and then depositing those transformed parcels back into other dynamical quantities. Any process being regarded as an external source or sink for parcels should instead be included as a dynamical quantity of the system.
3. Make a list of the *species* (the dynamical quantities) involved in the model.
  - Assign a variable name to each species.
  - Draw a box with the variable name in it for each species.
4. Make a list of the *interactions* (the different processes that define how the values of the species change in time) involved in the model.
  - Assign variable names to the properties associated with each interaction.
    - Identify which species are *sources* for the interaction (having parcels taken away by the interaction at each time step) and which are *targets* (receiving parcels).
    - Determine what size of parcel is drawn from each source species by each application of the interaction. These numbers are called the *source weights*.
    - Determine what size of parcel size is delivered to each target species by each application of the interaction. These numbers are called the *target weights*.
    - Determine what this interaction contributes to the fractional rate of change for each parcel of source species (*e.g.*, per capita change per hour, or the time derivative of the logarithm). In particular, how does the fractional rate of change scale with the values of each species in the system? (A lack of scaling—a numerical constant—is common in many models.) This functional dependence of the rate on the species is called the *interaction function*. It is based on the modeler’s empirical knowledge of the process and the system.
  - Draw a point for each interaction and label it with the interaction function.
  - Connect each interaction to its target and sources species.
    - Draw an arrow from each source species box to the interaction dot. Label it with that species’s source weight unless the weight is 1.
    - Draw an arrow from the interaction dot to each target species box. Label it with that species’s target weight unless the weight is 1.

Summarizing, each species is represented by a box with the variable name inside. The interaction is represented by a small dot labeled by the interaction strength (or interaction function, if it is not a constant). All source species for the interaction get an arrow from the box to the dot. All target species get an arrow from the dot to the box. If some of the source species contribute more than others, then the source arrows may be labeled with a weighting factor. If the targets do not receive equal fractions, or if the targets receive a different amount than is output from the sources, then the target arrows may be labeled with a weighting factor. Any unlabeled arrows are assumed to have weight 1.

The weights and interaction functions could be functions of time, or of the dynamical species values, or of external parameters, but in practice they will often be constants. However, the interaction function in particular will sometimes reflect a nontrivial functional dependence on the species values, for example being inversely proportional to the sum of all the species values. Weights may be pure numbers or they may have units, for example, “hares consumed per lynx birth” in a predator-prey system. Also, there is no requirement that weights into and out of an interaction be in balance: an interaction may represent a net gain of parcels, for example. Further discussion of the interpretation of weights and interaction functions is given in S2 Appendix.

Ultimately, a model with *M* species and *N* interactions will result in a network with *M* + *N* nodes: *M* boxes and *N* dots. Each box connects to some dots by a weighted directed edge outgoing to the dot for source boxes and incoming to the box for target boxes. Boxes cannot connect to boxes. Dots cannot connect to dots. Boxes are labeled with the species name. Dots are labeled with the interaction rate. All dots must have at least one incoming and one outgoing edge. All edges must terminate at one end on a box and at the other on a dot. “Loose ends” are not allowed. “Direct feeds” from one box to another—that is, transformations of a species without causation from an interaction—are not allowed. “Splits and merges” which connect dots without an intermediate box are not allowed.

### OFFL schema: from a diagram to ordinary differential equations

The interpretation of a given diagram as an ODE proceeds by the following rules:

- A network with *M* + *N* nodes in which *M* nodes are labeled as dynamical variables *X*^*i*^(*t*) (*i* ∈ {0…*M* − 1}) and the remaining *N* nodes are labeled with interaction functions *f*_*a*_ (*a* ∈ {0…*N* − 1}) will represent a system of *M* first order ODEs, *i.e.* a system of the form *dX*^*i*^/*dt* = *F*^*i*^(*X*) for some set of functions *F*^*i*^ to be determined as described below.
- The species nodes and interaction nodes are connected by directed labeled edges. For each interaction *f*_*a*_, note the number *p*_*a*_ of outgoing *target edges* and the number *q*_*a*_ of incoming *source edges*.
- Construct the array *α*_*a*,*m*_ which is the weight on the *m*^th^ target edge of the *a*^th^ interaction and the array *β*_*a*,*n*_ which is the weight on the *n*^th^ source edge of the *a*^th^ interaction.
- Also construct the *target array*
  $$\begin{array}{c} t_{b,m}^{i}=\left\{\begin{array}{cc} 1 & \text{if}\;X^{i}\;\text{is}\;\text{the}\;\text{target}\;\text{of}\;\text{the}\;m^{\text{th}}\;\text{edge}\;\text{of}\;\text{interaction}\;a\\ 0 & \text{otherwise} \end{array}\right. \end{array}$$ (1)
  and *source array*
  $$\begin{array}{l} s_{b,m}^{i}=\{\begin{array}{ll} 1 & \text{if }X^{i}\text{ is the source of the }m^{\text{th}}\text{ edge of interaction }a\\ 0 & \text{otherwise} \end{array}. \end{array}$$ (2)
- Then,
  $$\begin{array}{c} \frac{dX^{i}}{dt}=\sum_{a=0}^{N-1}\left[\left(\sum_{m=0}^{p_{a}-1}\alpha_{a,m}t_{a,m}^{i}-\sum_{n=0}^{q_{a}-1}\beta_{a,n}s_{a,n}^{i}\right)\;f_{a}\prod_{\ell =0}^{q_{a}-1}\left(\sum_{j=0}^{M-1}s_{a,\ell }^{j}X^{j}\right)\right]. \end{array}$$ (3)

This is the central result: an ODE which can be solved by standard techniques. Note that care should be taken not to confuse the time variable *t* and the target array $t_{b,m}^{i}$.

It is interesting to note that different diagrams can result in the same ODE, but the model interpretation of those diagrams as “systems of processes that redistribute transformed parcels among species” could be different. In the language of mathematical physics, transformations of model quantities (such as elements of the diagram) which leave the behavior of the model (that is, Eq 3) unchanged are known as “symmetries” of the system. Symmetries generally indicate a redundancy of description in the model and carry interesting qualitative consequences for the system’s dynamics, such as the existence of conserved quantities [11–13]. For example, in electrical circuit networks, the modeler’s freedom to choose the ground point at which voltage is zero is related in a deep way to the conservation of electric charge, which in turn leads to Kirchhoff’s laws for electrical currents: powerful relationships which constrain the possible behaviors of quantities in the network [14]. Though intriguing, for the sake of clarity we will not further explore these concepts in the current manuscript.

### Organizing OFFL: From a diagram to a database representation

**Fig 2:**
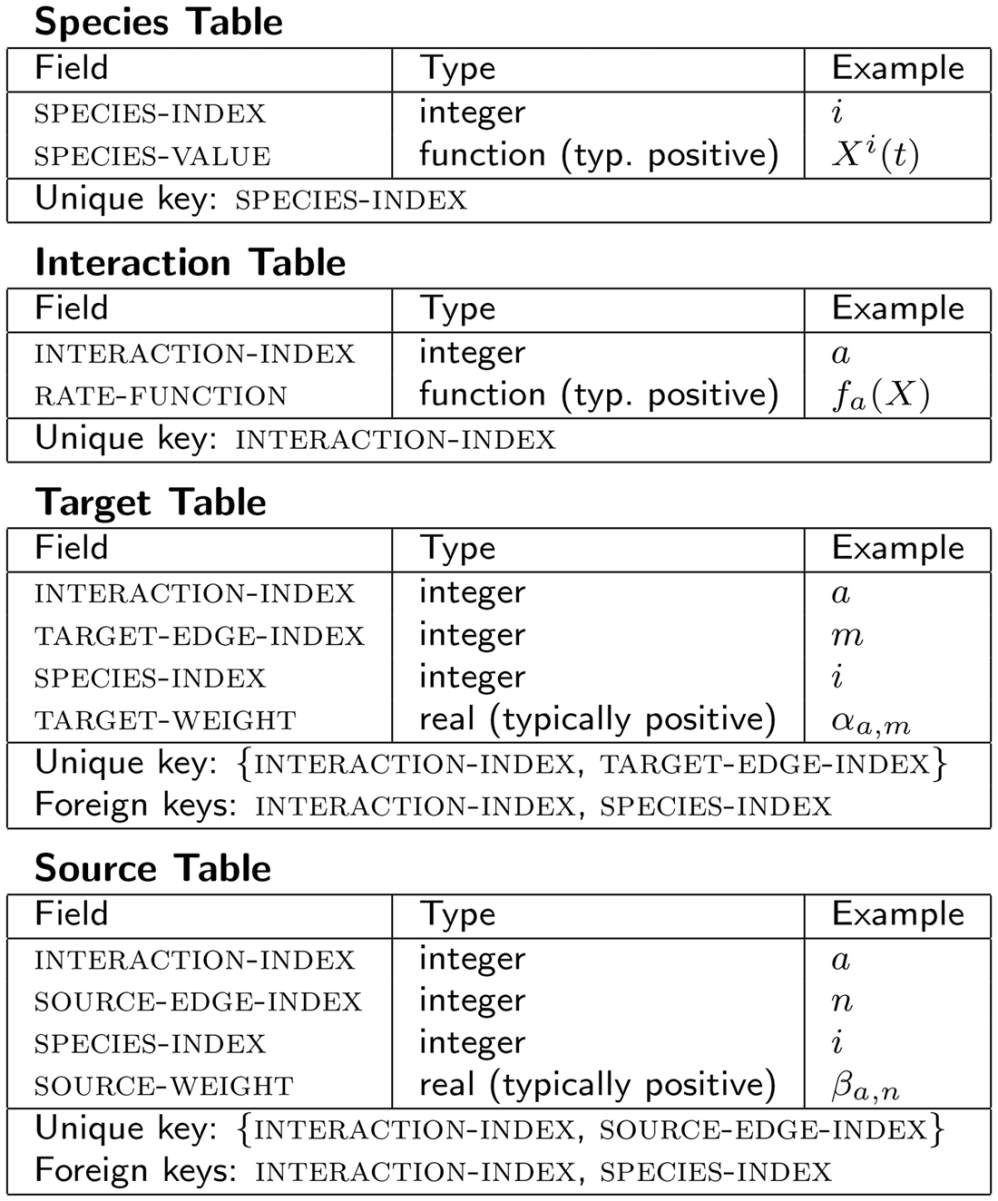
Database representation of an OFFL model. The four tables which represent OFFL diagrams as a relational database. The “Example” columns refer to quantities as they appear in Eq (3).

The various quantities described in the previous section which specify the diagram also define a relational database consisting of four tables: one each for the species nodes, the interaction nodes, the source edges, and the target edges. We prove this claim by explicit construction of the tables as shown in Fig 2.

In the language of relational databases, the source table and target table act as linking tables between the species table and interaction table.

Each table could of course be decorated with additional fields, like textual descriptions of the information being represented, or fields which support technical execution within a particular software implementation. For example, a key field consisting of a concatenation of the interaction-index and edge-index fields could be added to the target and source tables. Or, a new table uniquely keyed by node-index and the time variable could be used to keep track of the changing value of species-value, instead of dynamically updating the field in the node table.

The elements of an OFFL flow diagram and its database representation are in exact one-to-one correspondence, so the procedure given above for generating a diagram also applies to generating the database, and either the diagram or the database can be used equivalently in generating Eq (3). The database representation is readily stored and interpreted by a computer, making the generation and subsequent solution of Eq (3) for a given system a directly automatable process.

### Examples of simple processes as OFFL models

Many examples of practical importance involve only the linear processes of exponential growth and death along with bilinear (that is, nonlinear through the product of two variables) consumer-resource processes. As the core building blocks of many complex models, we consider these three processes here in some detail.

**Fig 3:**
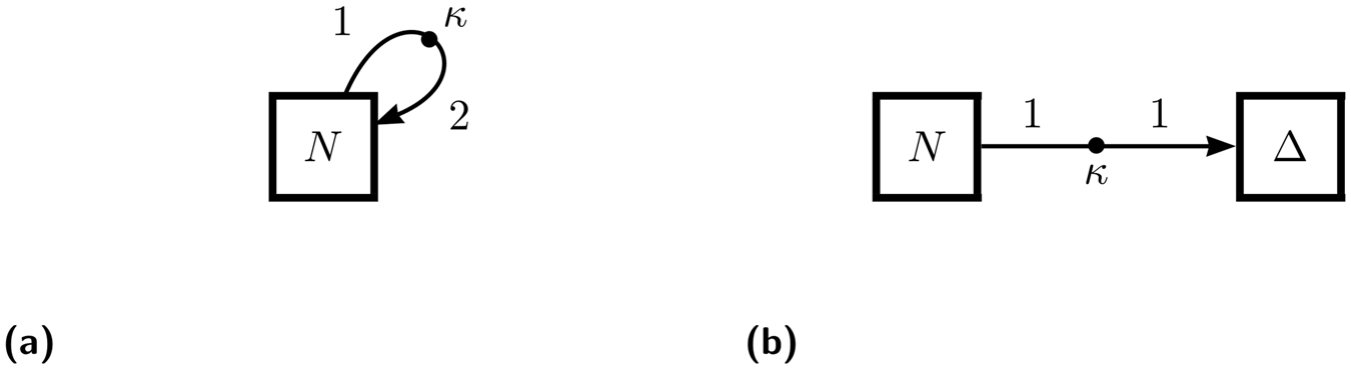
Exponential growth and death in the OFFL schema. (a) Growth is shown as positive feedback process. (b) Death is shown as a change of state.

Despite the processes of exponential growth (as shown in Fig 1d) and death being represented with the same ODE, differing only by the sign of the coefficient, the biological processes which give rise to these phenomena are quite different. Appropriately, they are represented quite differently as flow diagrams. In both processes, a parcel is drawn from the population *N* at rate *κ*. In growth, that parcel is replicated (say, duplicated, as shown in Fig 3a) and returned to the population. In death, however, the parcel is simply moved from a live state population to a dead state population Δ, as shown in Fig 3b. Thus, growth is given as *dN*/*dt* = *k*(−1 + 2)*N* = *κN*, while death is given by the system *dN*/*dt* = −*κN* and *d*Δ/*dt* = +*κN*.

The formal grammar of OFFL diagrams does not allow an edge to end without a target node. Therefore, the existence in Fig 3b of the target species Δ and its dynamical equation is required, but the dynamical equation for *N* remains independent of Δ and can be solved without the solution for Δ. The existence of such auxiliary variables—representing an external reservoir whose dynamics are unimportant to the system variables—is typical for OFFL models of “open” systems that exhibit processes similar to death, immigration, or emigration: processes that might be represented in a less formal schema by lines that enter or leave the diagram from nowhere. On the other hand, a process like death could perhaps be modeled instead as a spontaneous eradication rather than as a change of state. In this case, the diagram for the death process would take same form as for the growth process as in Fig 3a, except with a 0 instead of a 2 on the target edge, resulting in the single equation *dN*/*dt* = −*κN* and no auxiliary variable Δ representing a death state.

**Fig 4:**
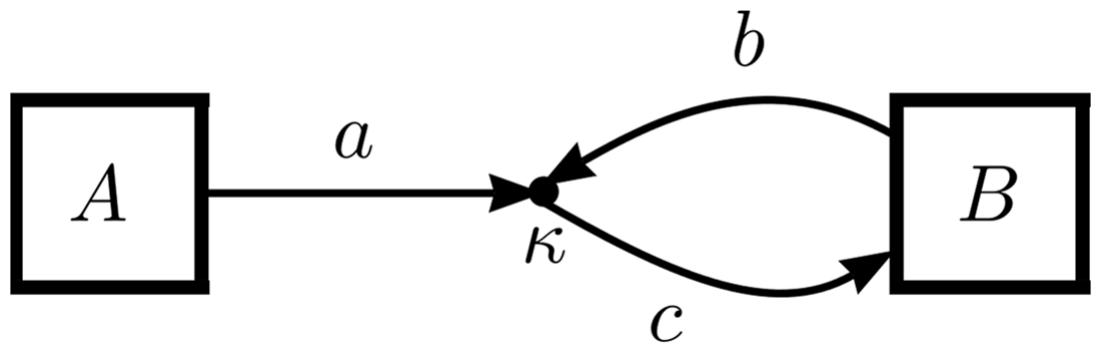
OFFL diagram for the most general two-species consumer-resource process.

The two-species consumer-resource interaction is perhaps the simplest nonlinear process appearing in the types of models under consideration here. One notable example, the SI epidemiological model, has already been presented in Fig 1b. The most general version of the process, in which *a* parcels of species *A* combine with *b* parcels of species *B* at rate *κ* to become *c* parcels of species *B* is shown in Fig 4. Its associated ODE is

dA/dt=-aκAB (4a)

dB/dt=(c-b)κAB. (4b)

Note, if the number of parcels incoming to the interaction equals those outgoing (*a* + *b* = *c*) as in the SI system, then $\frac{d}{dt}\left(A+B\right)=0$, and the total population is therefore constant. Otherwise, the consumer-resource process will lead to overall growth or death of the population.

## Discussion

In this section, we will discuss two slightly more complex examples. Both are established models whose usage is well understood in several scientific communities. This provides an opportunity to demonstrate how OFFL works and affirms that it can recapitulate results from well characterized biological problems. Additional examples will be given at http://modeling.mit.edu.

### Susceptible-Infected-Recovered (SIR) system

In an epidemiological SIR system [15], susceptibles *S* contact the infected *I* at a rate *c* (say, encounters per infected per day) of which a fraction *a* of contacts lead to new infections, while the infected spontaneously recover at a rate *ρ* (say, recoveries per infected per day), becoming the resistant population *R*. The specific system considered here also includes the waning of immunity at rate *σ* (losses per resistant per day), returning the resistant back to the susceptible population; death rates *d* and *δ* (deaths per person per day) for each species; and immigration of new susceptibles at rate *θ* (susceptibles per day).

**Fig 5:**
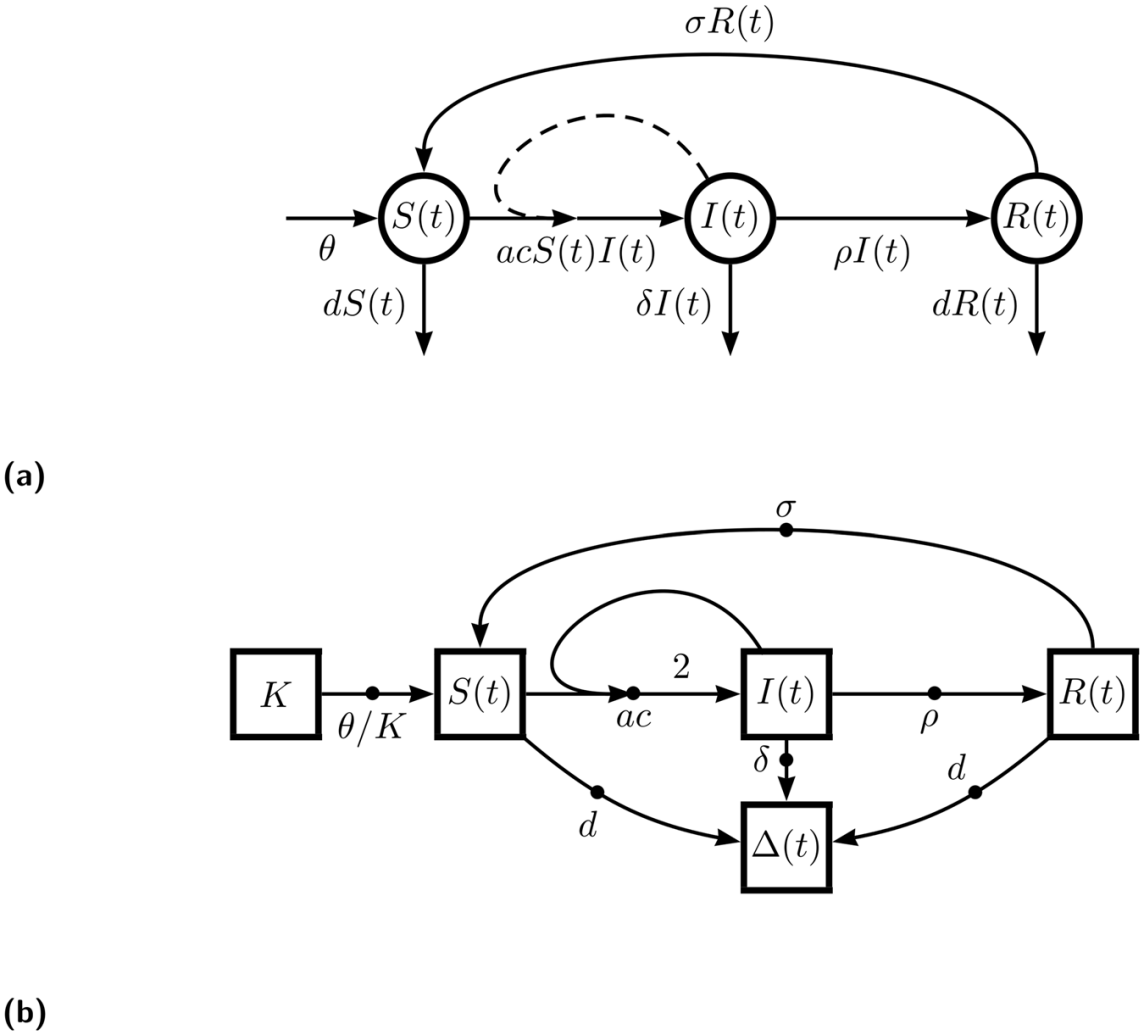
A simple epidemiological SIR model. Contact between susceptibles *S* and infected *I* leads to new infections, while simultaneously the infected can spontaneously recover and become resistant to the infection *R*. (a) An informal flow diagram for the SIR model as shown, for example, in Figs 3–9 of Ref. [7]. (b) The OFFL version of SIR. The new “dead pool” species Δ is the target of the death interactions. The new external “community pool” species *K* is the source of the immigration interaction. The model has 5 species and 7 interactions.

The SIR system is ideally suited for modeling as a flow diagram. Fig 5 shows a side-by-side comparison of two different schema representations of this SIR model: a traditionally informal flow diagram and an OFFL diagram. The OFFL diagram is generated by starting from the above description of the system and following the steps given in the Methods section.

Given the diagram, the rules leading to Eq (3) then illustrate how to generate a set of ODEs, which proceeds as follows. We first identify all the species and name them: along with the three dynamic species *S*, *I*, and *R*, we also add a “community pool” species *K* which is the source for external immigration and a “dead pool” species Δ which is the target for death interactions. Next, reasoning through the description of the model given above, we see there are seven interactions in the model: the death of each of the three dynamical species (moving a unit parcel from *S*, *I*, or *R* to Δ with rate *δ* or *d*), spontaneous recovery (moving a unit parcel from *I* to *R* with rate *ρ*), loss of immunity (moving a unit parcel from *R* to *S* with rate *σ*), infection (in which a parcel of *S* interacts with a parcel *I* at rate *ac* to become two parcels of *I*), and immigration (moving a unit parcels from *K* to *S*). In immigration, *θ*—the absolute rate of change to *S*—is presumed by the model to be constant even as the number of available parcels in the *K* population varies, so it must the case that the strength of the process which attracts parcels from *K* to *S* must vary as ∼1/*K*, specifically *θ*/*K*. The dynamics of *S*, *I*, and *R* are then independent of the dynamics of *K*.

The resulting ODE is then

dS/dt=-acSI+σR-dS+θ (5a)

dI/dt=acSI-ρI-δI (5b)

dR/dt=+ρI-σR-dR (5c)

dK/dt=-θ (5d)

dΔ/dt=dS+δI+dR. (5e)

Eqs (5a), (5b) and (5c) are the essential dynamics of the system. These are the equations which would result from a standard reading of the informal diagram in Fig 5a. As discussed at the end of the Methods section, OFFL results in two additional equations for *K* and Δ, but these are trivial additions to the dynamics which can be ignored when solving for *S*, *I*, and *R*.

Note that to maintain realism in the model, the initial condition on *K* should be sufficiently large that *K* will not change appreciably during the run time *τ* of the model, *K*(0) ≫ *θτ*. Or, alternatively, one could force *K* to be constant *dK*/*dt* = 0 by any number of ways while leaving the rest of the ODE unchanged, such as setting the immigration source weight to 0.

### Lotka-Volterra predator-prey system

**Fig 6:**
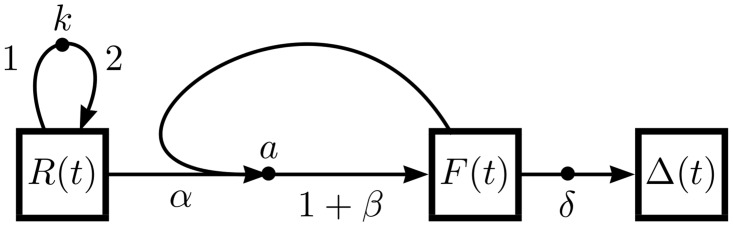
Lotka-Volterra flow diagram. The Lotka-Volterra model is the simplest predator-prey model. A prey species *R* (“rabbits”) reproduce spontaneously at rate *k* while a predator species *F* (“foxes”) consume *α* rabbits at rate *a* and convert them to *β* new foxes. Foxes move spontaneously at rate *δ* to the dead pool Δ.

The Lotka-Volterra system is one of the defining models in ecology, and has served as a basis for understanding predator-prey dynamics for many years [16–18]. Although superseded by more modern models in ecological research, Lotka-Volterra remains a classic application of mathematical modeling. The OFFL treatment of Lotka-Volterra is shown in Fig 6, with the system itself described in the figure caption. Following the steps leading to Eq (3), Fig 6 renders to the following equations:

dR/dt=kR-aαRF (6a)

dF/dt=aβRF-δF (6b)

dΔ/dt=δF. (6c)

As discussed in the Methods section, modeling the death of the predator species *F* again involves the addition of an auxiliary species Δ whose dynamics are ultimately irrelevant to the other dynamical species.

In the language of mathematical ecology, the “functional response” of the model in this case is so-called type I. That is, the rate of prey consumption is linear in prey population [19], indicating that predators do not eat any less often as they consume prey: they never get “full”. Changing to a type II functional response—which saturates to a constant as the predators get full—is as simple as changing *a* in Fig 6 to a function of *F* and *R*, say *a*(*F*, *R*) = *a*/(1 + *haR*) for some “handling time” *h*. This easy extensibility of the simple model into a more complicated one is typical of the OFFL schema.

## Conclusions

The goal of this manuscript was to describe a framework which puts the power of mathematical modeling into the hands of content experts who might otherwise avoid the use of highly mathematical tools. We focused on a particular framework that manages systems well-described by ODEs (“interaction networks”), but the general program should be considered open for tools which address other mathematical methods (such as stochastic systems) in a similar spirit. Specifically, we have attempted the following:

- Introduce the OFFL schema, explaining both how it works and its theoretical underpinnings
- Highlight differences between OFFL and related approaches to modeling biological systems which also use flow diagrams and ODEs
- Illustrate how OFFL might offer advantages in modeling dynamical systems

Mathematically, we have shown that the broad class of dynamical systems under consideration here can be represented in three complementary ways: a weighted directed graph (a network), a system of ODEs, and a relational database. While fundamentally interesting and a clear opportunity for future research into the properties of these systems, further mathematical study of this observed triality is beyond the scope of the present manuscript and will be left for future communications.

Algorithmization of the model building process naturally lends itself to a software engineering implementation. One could imagine a graphical front end that allows a would-be modeler to draw an OFFL diagram intuitively—based, for example, on the modeler’s observations of infectious disease dynamics in the field—while all the mathematics is carried out behind the scenes. This can put the modeling of even complicated outbreaks into the hands of front line medical personnel and public health officials while requiring little mathematical expertise. Development of such a software implementation of OFFL as a free web application [20] is presently underway and will be the subject of a future report. Such a software solution could also enable improved instructional methods in and appreciation for the value of quantitative modeling in the biological, public health, and medical student communities.

Because the OFFL approach lends itself so readily to automation, it is tempting to compare it to the myriad of existing software packages and simulators that model complex systems in biology. Before making any direct comparisons, however, we should reiterate that this manuscript is intended as neither a user manual nor a rationale for a pending software package. Instead, OFFL is designed and presented as an epistemic framework for understanding how modeling works in biological systems. The true goal of this approach, then, is not to make the act of simulating a dynamic system easier through software, but rather to support the systematic use of systems thinking and the iterative mental processes of model building (and, perhaps, modeling education).

We might even say that OFFL could be compatible with existing software packages in the systems biology and epidemiology communities. We suggest that users of these software tools make use of the OFFL framework in constructing models (perhaps through “pen and paper” methods) before using any given package or simulator. Additionally, for instructors and students, OFFL is a way of introducing modeling concepts prior to using any software package.

Nevertheless, a modeler might choose an OFFL-based software tool for a variety of reasons. Existing software packages for modeling in systems biology and related fields tend to have applications focused on specific classes of problems, such as VCell[21] (cell biology, biochemistry), CellDesigner[22–24] (biochemistry and gene regulation), and EpiModel[25] (modeling of epidemics). In this sense, OFFL differs in its purpose and fungibility. It can be used for any given systems-style model in ecology, evolution, epidemiology, chemical kinetics, or even social sciences. Because of this, OFFL might be compared to software packages like STELLA[26] or Berkeley Madonna[27], both highly developed and well-regarded systems simulation software packages whose developers also have a mind towards making modeling accessible to novice users and promoting widespread usage of systems thinking. But while STELLA and Berkeley Madonna have many potential applications (including in social sciences and business), they are not free packages. Again, it may be possible to implement the OFFL schema within any of the above software tools, but for an OFFL-based software tool to not miss the point of using the OFFL schema, it should take care to present a user interface which only requires graphical manipulation of the species and interactions of the model, avoiding any user intervention requiring advanced mathematical or computational training.

Several authors have noted the importance of universal standards for the representation and transmission of information about models in systems biology, resulting in successful projects such as the Systems Biology Markup Language (SBML) [28] and Systems Biology Graphical Notation (SBGN) [29]. OFFL can work within these modern standards; in particular, we note that OFFL diagrams are readily represented using the entity pool nodes, process nodes, consumption arcs, and production arcs of the SBGN Process Description language (SBGN-PD) [30]. (However, modulation arcs from SBGN-PD do not have a direct interpretation under the rules of OFFL, which requires that modulations such as catalysis be either explicitly modeled or represented in an interaction function.) However, we must again emphasize that OFFL differs from strictly symbolic notational frameworks like SBGN because it is more than a visual standard for depicting qualitative biological relationships. Instead, OFFL’s elements have dedicated mathematical definitions, and consequently, can be used to model quantitative relationships between actors (biological, social, or other), not simply illustrate them.

In the end, improving the community’s general mathematical literacy is a daunting task that requires commitment and investment from educators, politicians, and active researchers alike. That said, even a small pedagogical breakthrough can go a long way—by being engaged, improved upon, and modified—towards bridging gaps between complicated mathematics and real world applications.

The OFFL modeling approach presented here aims to improve our understanding of dynamical systems modeling, a tool that is increasingly useful to both practitioners of science and citizen-scientists in an increasingly complex world.

## Supporting Information

S1 Appendix Definitions of terms introduced as jargon of the OFFL schema. (PDF)

S2 Appendix Discussion of interaction functions and the meaning of edge weights in an OFFL model. (PDF)

## References

[1] Kuhn T. The Structure of Scientific Revolutions. University of Chicago Press; 1962.

[2] Pachter L. The two cultures of mathematics and biology [Internet]. Bits of DNA; 30 December 2014 [cited 5 Nov 2015]. Available from: https://liorpachter.wordpress.com/2014/12/30/the-two-cultures-of-mathematics-and-biology/.

[3] Fawcett TW, Higginson AD. Heavy use of equations impedes communication among biologists. Proceedings of the National Academy of Sciences of the United States of America. 2012;109(29):11735–11739. Available from: http://www.pnas.org/content/109/29/11735.abstract. 10.1073/pnas.1205259109 22733777 PMC3406806

[4] Chitnis N, Smith TA. Mathematical illiteracy impedes progress in biology. Proceedings of the National Academy of Sciences of the United States of America. 2012;109(45):E3055 Available from: http://www.pnas.org/content/109/45/E3055.short. 10.1073/pnas.1213115109 22996332 PMC3494925

[5] Feinberg M. Lectures on Chemical Reaction Networks [Internet]; 1979 [cited 5 Nov 2015]. Available from: https://crnt.osu.edu/LecturesOnReactionNetworks.

[6] American Association of Medical Colleges, Howard Hughes Medical Institute. Scientific Foundations for Future Physicians: Report of the AAMC-HHMI Committee [Institutional report]; 2009. Available from: https://www.aamc.org/download/271072/data/scientificfoundationsforfuturephysicians.pdf.

[7] Otto SP, Day T. A Biologist’s Guide to Mathematical Modeling in Ecology and Evolution. 1st ed Princeton, New Jersey: Princeton University Press; 2007.

[8] Barabási AL. Linked: The New Science of Networks. New York: Perseus Books Group; 2002.

[9] Watts D. Six Degrees: The Science of a Connected Age. New York: W. W. Norton & Company; 2002.

[10] Meadows DH, Meadows DL, Randers J. Limits to Growth: The 30-year Update. White River Junction, VT: Chelsea Green Pub. Co; 2004.

[11] Noether E. Invariante variationsprobleme. Nachrichten von der Königlichen Gesellschaft der Wissenschaften zu Göttingen, mathematisch-physikalische Klasse. 1918;1918:235–257.

[12] Park D. Resource Letter SP-1 on Symmetry in Physics. American Journal of Physics. 1968;36(7):577–584. Available from: http://scitation.aip.org/content/aapt/journal/ajp/36/7/10.1119/1.1975017. 10.1119/1.1975017

[13] Rosen J. Resource letter SP-2: Symmetry and group theory in physics. American Journal of Physics. 1981;49(4):304–319. Available from: http://scitation.aip.org/content/aapt/journal/ajp/49/4/10.1119/1.12504. 10.1119/1.12504

[14] Kirchhoff S. Ueber den Durchgang eines elektrischen Stromes durch eine Ebene, insbesondere durch eine kreisförmige. Annalen der Physik. 1845;140(4):497–514. Available from: 10.1002/andp.18451400402. 10.1002/andp.18451400402

[15] Kermack WO, McKendrick AG. A Contribution to the Mathematical Theory of Epidemics. Proceedings of the Royal Society of London A: Mathematical, Physical and Engineering Sciences. 1927;115(772):700–721. Available from: http://rspa.royalsocietypublishing.org/content/115/772/700. 10.1098/rspa.1927.0118

[16] Lotka AJ. Analytical Note on Certain Rhythmic Relations in Organic Systems. Proceedings of the National Academy of Sciences of the United States of America. 1920;6(7):410–415. Available from: http://www.pnas.org/content/6/7/410.short. 10.1073/pnas.6.7.410 16576509 PMC1084562

[17] Volterra V. Variazioni e fluttuazioni del numero d’individui in specie animali conviventi. Memoria della Regia Accademia Nazionale dei Lincei. 1926;2.

[18] Volterra V. Fluctuations in the Abundance of a Species considered Mathematically. Nature. 1926;118 Available from: http://www.nature.com/nature/journal/v118/n2972/abs/118558a0.html. 10.1038/118558a0

[19] Holling CS. The Components of Predation as Revealed by a Study of Small-Mammal Predation of the European Pine Sawfly. The Canadian Entomologist. 1959 5;91:293–320. Available from: http://journals.cambridge.org/article_S0008347X00072564. 10.4039/Ent91293-5

[20] Caruso W, Squires C. OFFlMaker [Software]. Open source; Anticipated beta release 2016. Available from: http://modeling.mit.edu/offlmaker.

[21] Schaff J. VCell [Software]. University of Connecticut Health Center: Center for Cell Analysis & Modeling; 1997. Available from: http://www.nrcam.uchc.edu.

[22] Funahashi A, Kitano H, Jouraku A, Kikuchi N, Ghosh S, Matsuoka Y. CellDesigner [Software]. The Systems Biology Institute; 2003. Available from: http://www.celldesigner.org/.

[23] Funahashi A, Tanimura N, Morohashi M, Kitano H. CellDesigner: a process diagram editor for gene-regulatory and biochemical networks. BIOSILICO. 2003;1(5):159–162. Available from: http://www.sciencedirect.com/science/article/pii/S1478538203023709. 10.1016/S1478-5382(03)02370-9

[24] Funahashi A, Matsuoka Y, Jouraku A, Morohashi M, Kikuchi N, Kitano H. CellDesigner 3.5: A Versatile Modeling Tool for Biochemical Networks. Proceedings of the IEEE. 2008 8;96(8):1254–1265. Available from: http://ieeexplore.ieee.org/xpl/articleDetails.jsp?arnumber=4567412. 10.1109/JPROC.2008.925458

[25] Jenness, SM, Goodreau, SM, Morris M. EpiModel: Mathematical Modeling of Infectious Disease [Software]; 2016. R Package Version 1.2.5. Available from: http://www.epimodel.org/. 10.18637/jss.v084.i08 PMC5931789 29731699

[26] Richmond B. STELLA [Software]. Lebanon, NH: isee systems; 1985 Available from: http://www.iseesystems.com/softwares/Education/StellaSoftware.aspx.

[27] Macey R, Oster G, Zahnley T. Berkeley Madonna [Software]. Berkeley, CA; 1993 Available from: http://www.berkeleymadonna.com/.

[28] Hucka M, Finney A, Sauro HM, Bolouri H, Doyle JC, Kitano H, et al The systems biology markup language (SBML): a medium for representation and exchange of biochemical network models. Bioinformatics. 2003;19(4):524–531. Available from: http://bioinformatics.oxfordjournals.org/content/19/4/524.abstract. 10.1093/bioinformatics/btg015 12611808

[29] Le Novere N, Hucka M, Mi H, Moodie S, Schreiber F, Sorokin A, et al The systems biology graphical notation. Nature Biotechnology. 2009;27(8):735–741. Available from: http://www.nature.com/nbt/journal/v27/n8/suppinfo/nbt.1558_S1.html. 10.1038/nbt.1558 19668183

[30] Moodie S, Le Novere N, Demir E, Mi H, Villeger A. Systems Biology Graphical Notation: Process Description language Level 1. Nature Precedings. 2011;Available from: http://precedings.nature.com/documents/3721/version/4. 10.1038/npre.2011.3721.4 26528561

