# Supplementary material for: OFFl Models: Novel Schema for Dynamical Modeling of Biological Systems
Source: PLoS One. 2016 Jun 7;11(6):e0156844. doi: 10.1371/journal.pone.0156844 (PMC4896432; doi:10.1371/journal.pone.0156844)
Supplement: S1 Appendix — (PDF) [file pone.0156844.s001.pdf]

S1 Appendix. Supplementary material for "OFFL Models: Novel Schema for Dynamical Modeling of Biological Systems"

C. Brandon Ogbunugafor*† Sean P. Robinson‡

Definitions of terms introduced as jargon of the OFFL schema are as follows.

interaction network The type of system that the OFFL schema can describe, modeled by a system of coupled ODEs. Parcels of various dynamical species in the model are transformed into parcels of other species at rates determined by a set of interaction rules that depend on the population values of the species.

parcel The unit of a species which participates in each application of an interaction. Indicated on an OFFL diagram by the weight labels.

species The model representation of the dynamical quantities of a system. Species are changed over time in response to the other species by the interactions. Represented in OFFL by a box labeled with the species name.

interaction The model representation of the processes of a system which cause the changes in the species. Represented in OFFL as a dot labeled by the interaction function.

source Species from which parcels are drawn by an interaction. Represented in OFFL as a directed edge pointing from a species to an interaction, labeled by a weight.

target Species to which parcels are delivered by an interaction. Represented in OFFL as a directed edge pointing from an interaction to a species, labeled by a weight.

weights Label on an edge of an OFFL diagram indicating the parcel size for the species drawn out or delivered by the interaction.

interaction function The rate of change per unit of source species (exclusive of per-species weighting) induced in each species involved in an interaction. Used to label the interaction dots in an OFFL diagram.

The following terms are not part of the OFFL schema, but are included here to clarify their meaning as used in this document.

system The subject under study which physically exists in the real world (or possibly a hypothetical world) and can be probed by observation and experiment.

model A representation of a way of understanding the system. May be mathematical, taxonomical, *etc.* Essentially all modes of understanding systems can be considered models, and essentially all models describe only a fraction of the system's behavior or composition. The value of a model is judged by how faithfully it represents the aspects of the system which are of interest to the modeler. In OFFL, the system is represented in three mathematically equivalent ways: a diagram, a database, and a set of ODEs.

process Mechanism by which a system changes in time. In OFFL, processes are represented as interactions which move parcels between species.

*Department of Organismic and Evolutionary Biology, Harvard University
†Department of Biology, University of Vermont
‡Department of Physics, Massachusetts Institute of Technology
