# Supplementary material for: OFFl Models: Novel Schema for Dynamical Modeling of Biological Systems
Source: PLoS One. 2016 Jun 7;11(6):e0156844. doi: 10.1371/journal.pone.0156844 (PMC4896432; doi:10.1371/journal.pone.0156844)
Supplement: S2 Appendix — (PDF) [file pone.0156844.s002.pdf]

S2 Appendix. Supplementary material for "OFFL Models: Novel Schema for Dynamical Modeling of Biological Systems"

C. Brandon Ogbunugafor*† Sean P. Robinson‡

Discussion of interaction functions and the meaning of edge weights in an OFFl model

The interpretation of an OFFL diagram follows the simple rule that an arrow leaving a box reduces the value of that species while an arrow entering a box increases it. The interaction function determines the interaction's contribution to the fractional rate of change relative to the source terms for all species connected to the interaction. That is, the absolute rate of change due to the interaction is the product of the interaction strength and the values of all the source species. Each species connected to the interaction then gets a contribution to its instantaneous rate of change given by the product of the above absolute rate with the species's weight factor for that interaction, keeping in mind that the contribution to source terms is negative. So, the total rate of change for a species is a sum of terms, one for each interaction affecting the species.

The source and target weights can be thought of as a kind of conversion factor between species. For example, if a model describes an organism consuming some amount of food before reproducing, the source edge from the food species to the reproduction interaction would have a weight indicating how much food must be consumed to produce each new organism (that is, for each application of the interaction function), while the source and target edges between the reproduction interaction and the organism will be weighted with values 1 and 2, respectively, indicating that the reproduction process results in two organisms for each initial organism participating (binary fission). (The diagram for this model would be similar to the consumer-resource process shown in Fig. 4, with A as the food, B as the organism, a as the quantity of food to be consumed, $b = 1$, and $c = 2$.) In this case, the weight of 2 on the target edge can be viewed as kind of amplification or gain factor, which is a valid alternative point of view for understanding the source and target weights. This illustrates the point that interactions need not be "conservative" or "unity gain" in OFFL: a unit parcel of source species need not be converted into a unit parcel of target species.

*Department of Organismic and Evolutionary Biology, Harvard University
†Department of Biology, University of Vermont
‡Department of Physics, Massachusetts Institute of Technology
